# Novel and reported compensatory mutations in *rpoABC* genes found in drug resistant tuberculosis outbreaks

**DOI:** 10.3389/fmicb.2023.1265390

**Published:** 2024-01-08

**Authors:** Derek Conkle-Gutierrez, Sarah M. Ramirez-Busby, Bria M. Gorman, Afif Elghraoui, Sven Hoffner, Wael Elmaraachli, Faramarz Valafar

**Affiliations:** ^1^Laboratory for Pathogenesis of Clinical Drug Resistance and Persistence, San Diego State University, San Diego, CA, United States; ^2^Department of Global Public Health, Karolinska Institute, Stockholm, Sweden; ^3^Division of Pulmonary, Critical Care, and Sleep Medicine, University of California, San Diego, San Diego, CA, United States

**Keywords:** tuberculosis, *Mycobacterium tuberculosis*, rifampicin, antibiotic resistance, compensatory mutations, fitness

## Abstract

**Background:**

Rifampicin (RIF) is a key first-line drug used to treat tuberculosis, a primarily pulmonary disease caused by *Mycobacterium tuberculosis*. RIF resistance is caused by mutations in *rpoB*, at the cost of slower growth and reduced transcription efficiency. Antibiotic resistance to RIF is prevalent despite this fitness cost. Compensatory mutations in *rpoABC* genes have been shown to alleviate the fitness cost of *rpoB*:S450L, explaining how RIF resistant strains harbor this mutation can spread so rapidly. Unfortunately, the full set of RIF compensatory mutations is still unknown, particularly those compensating for rarer RIF resistance mutations.

**Objectives:**

We performed an association study on a globally representative set of 4,309 whole genome sequenced clinical *M. tuberculosis* isolates to identify novel putative compensatory mutations, determine the prevalence of known and previously reported putative compensatory mutations, and determine which RIF resistance markers associate with these compensatory mutations.

**Results and conclusions:**

Of the 1,079 RIF resistant isolates, 638 carried previously reported putative and high-probability compensatory mutations. Our strict criteria identified 46 additional mutations in *rpoABC* for which no strong prior evidence of their compensatory role exists. Of these, 35 have previously been reported. As such, our independent corroboration adds to the mounting evidence that these 35 also carry a compensatory role. The remaining 11 are novel putative compensatory markers, reported here for the first time. Six of these 11 novel putative compensatory mutations had two or more mutation events. Most compensatory mutations appear to be specifically compensating for the fitness loss due to *rpoB*:S450L. However, an outbreak of 22 closely related isolates each carried three *rpoB* mutations, the rare RIF^R^ markers D435G and L452P and the putative compensatory mutation I1106T. This suggests compensation may require specific combinations of *rpoABC* mutations. Here, we report only mutations that met our very strict criteria. It is highly likely that many additional *rpoABC* mutations compensate for rare resistance-causing mutations and therefore did not carry the statistical power to be reported here. These findings aid in the identification of RIF resistant *M. tuberculosis* strains with restored fitness, which pose a greater risk of causing resistant outbreaks.

## Introduction

Rifampicin (RIF) is an important first-line drug used to treat tuberculosis (TB), a pulmonary disease caused by *Mycobacterium tuberculosis* (World Health Organization, 2022). TB is a global pandemic, with an estimated 10 million cases and causing 1.6 million deaths in 2021 (World Health Organization, 2022). Unfortunately, RIF resistance (RIF^R^) is prevalent. An estimated 450,000 TB cases were RIF^R^ in 2021, including 3.6% of new TB cases and 18% of previously treated TB cases (World Health Organization, 2022). RIF^R^ and multidrug resistant (MDR) TB require 6 to 20 months of treatment with second-line drugs (World Health Organization, 2022).

RIF^R^ in *M. tuberculosis* is principally due to point mutations in the RIF resistance-determining region (RRDR) (Telenti et al., 1993; Miller et al., 1994; Ramaswamy and Musser, 1998), an 81-bp region (codons 426–452 in *M. tuberculosis*, codons 507–533 in *E. coli*) of the gene *rpoB* (Jin and Gross, 1988), though two *rpoB* mutations outside the RRDR have also been shown to cause RIF resistance (World Health Organization, 2021a). The *rpoB* gene encodes the β-subunit of RNA polymerase (Jin and Gross, 1988). Mutations in the RRDR typically confer resistance by a change in the three-dimensional protein structure of the β-subunit, disrupting the RIF binding site (Nusrath Unissa et al., 2016). The strong association between mutations in the RRDR and RIF resistance has led to the development of molecular tests and WHO recommended diagnostic platforms, including GeneXpert MTB/RIF, Truenat (Penn-Nicholson et al., 2021), the line probe assays GenoType MTBDRplus VER 1 and 2 (Hain Lifescience, Germany), the Genoscholar NTM + MDRTB detection kit 2 (Nipro, Japan), and others (Saiki et al., 1989; Raja et al., 2005; World Health Organization, 2021b).

However, mutations in the RRDR have a fitness cost, slowing *M. tuberculosis* growth *in vitro* (Mariam et al., 2004; Gagneux et al., 2006) and in macrophage (Mariam et al., 2004). Despite the fitness cost, RIF^R^ strains continue to emerge, spread, and cause outbreaks (World Health Organization, 2020). This discrepancy may be due to compensatory mutations. Gagneux et al. observed that different *M. tuberculosis* strains with the same *rpoB* mutations had different fitness costs, and suggested the fitness costs were reduced in some strains by compensating mutations (Gagneux et al., 2006). Later, Gagneux and Comas et al. identified 12 high-probability compensatory mutations (HCMs) in *rpoA* and *rpoC*, which encode the $\beta^{\prime }$ and α subunits of RNA polymerase (Comas et al., 2012). These 12 HCMs were carried by polyphyletic and exclusively RIF^R^ RRDR-variant *M. tuberculosis* isolates, suggesting these HCMs were selected for in RIF^R^ isolates (Comas et al., 2012). Meanwhile in *Salmonella enterica*, several mutations in *rpoA* and *rpoC* have also been confirmed by mutagenesis to restore wild type growth *in vitro* to *rpoB* mutants (Brandis et al., 2012).

Compensatory mutations have been shown to increase the growth rate of RIF^R^ strains (Brandis et al., 2012; Comas et al., 2012), restore efficiency of transcription (Stefan et al., 2018), prevent reversion to wildtype(Brandis et al., 2012), and associate with increased transmission(Comas et al., 2012; de Vos et al., 2013; Li et al., 2016; Merker et al., 2018; Gygli et al., 2021). Though one contradicting study found no association between putative compensatory mutations and transmission cluster size among MDR-TB strains (Liu et al., 2018). Once the transmissibility question is fully settled, compensatory mutations might serve as markers warning which RIF^R^ strains are more likely to cause outbreaks. However, a key obstacle to this utility is uncertainty over which *rpoABC* mutations compensate, and which RIF^R^ markers they compensate for.

In this work, we sought to expand the set of putative compensatory mutations and determine which specific RIF^R^ markers they associate with. We investigated the sequences of *rpoABC* genes in 4309 clinical *M. tuberculosis* isolates, of which 1,079 were RIF^R^. This work identified 18 novel putative compensatory mutations. Additionally, both novel and previously reported putative compensatory mutations were found to be associated with the specific RIF^R^ marker *rpoB*:S450L, suggesting compensatory effects may be specific to particular *rpoB* mutations. These findings can help determine which RIF^R^ strains are compensated, which will both aid future studies in determining the role of compensation in transmission and eventually may serve as markers warning which resistant strains are most prone to causing outbreaks.

## Materials and methods

### Data availability statement

All supplemental tables are available at https://doi.org/10.5281/zenodo.7324837.

### Sample selection

We analyzed a total of 4,309 whole genome sequences. Of these 314 were sequenced on Pacific Bioscience’s (PacBio) Real-time Sequencer (RS) and RS II platforms (Bioproject: PRJNA353873). These isolates originated from Hinduja National Hospital (PDHNH) in Mumbai, India; the Phthisiopneumology Institute (PPI) in Chisinau, Moldova; the Tropical Disease Foundation (TDF) in Manila, Philippines; the National Health Laboratory Service of South Africa (NHLS) in Johannesburg, South Africa.

We also downloaded 3,995 whole genome sequences from NCBI’s Sequence Read Archive (SRA) database using SRA Toolkit’s fastqdump (Leinonen et al., 2011) (Bioprojects: PRJEB2221, PRJEB5162, PRJEB6276, PRJEB7281, PRJEB7727, PRJEB9680, PRJNA282721, PRJEB2138). All downloaded raw reads were sequenced on Illumina platforms. These genomes were isolated from patients originating from the UK, Sierra Leon, South Africa, Germany, Uzbekistan (Walker et al., 2015), and Russia (Casali et al., 2012, 2014; Chernyaeva et al., 2014).

### Drug susceptibility testing

The RIF susceptibility testing for the PacBio and Illumina sequenced isolates were described in Casali et al. (2012, 2014), Chernyaeva et al. (2014), Rodwell et al. (2014), Garfein et al. (2015), and Walker et al. (2015), respectively. Briefly, all samples were tested on the BACTEC MGIT960 platform and a rifampicin (RIF) critical concentration of 1 μg/mL. DST on all isolates was performed prior to the 2021 change in the recommended critical concentration, and thus used the higher previous critical concentration (Köser et al., 2021).

### Whole genome sequencing

The DNA sequencing protocol for the PacBio RS and RSII platforms was described previously (Torres et al., 2015). The sequencing protocol for genomes sequenced on Illumina Genome Analyzer, MiSeq, or HiSeq platforms were described previously (Casali et al., 2012, 2014; Chernyaeva et al., 2014; Walker et al., 2015).

### Alignment and variant calling

For 268 isolates sequenced on the PacBio RSI platform, raw reads were aligned to *M. tuberculosis* H37Rv reference strain (Genbank accession number NC_000962.3) utilizing SMRT Analysis’ BLASR with default parameters (v1.3) (Chaisson and Tesler, 2012). For variant calling, a custom software, PBHoover (Ramirez-Busby et al., 2018) (manuscript submitted), corrected aligned reads and called variants based on a maximum likelihood criterion. PBHoover determines the threshold number of supporting reads for each variant based on the sequencing depth, the type of mutation, and the per base per read for the specific mutation type and sequencing platform (Ramirez-Busby et al., 2018). The software was validated by comparing PBHoover variant calls with PacBio RSI sequencing data in 348 isolates to published variant calls made from targeted Sanger sequencing of the same isolates, showing 99.95% concordance between them (801,516/801,930 calls were concordant) (Ramirez-Busby et al., 2018).

For 46 isolates sequenced on the PacBio RSII platform and passing assembly quality control, whole genomes were assembled with HGAP as described previously (Modlin et al., 2020). The assembled genomes were reference quality passing stringent quality controls, including consensus polishing and circulurization (Modlin et al., 2020). The assembled genomes were aligned to H37Rv and variant called using dnadiff (v1.3) from the mummer suite (Kurtz et al., 2004). Variants were converted to VCF format using a custom script, mummer-snps2vcf.^1^

All Illumina short-read data were processed as follows: trimmomatic (v0.36) (Bolger et al., 2014) trimmed adapters from raw reads; bowtie2 (v2.2.4) (Langmead and Salzberg, 2012) aligned reads to the *M. tuberculosis* H37Rv reference sequence (Genbank accession NC_000962.3); SAMtools (v1.3.1) (Li et al., 2009) sorted, filtered out reads with a mapping quality of less than 20, and created an mpileup file for each isolate; VarScan2 (v2.3) (Koboldt et al., 2012) called and filtered variants with a minimum quality of 20, a minimum depth of 10, and strand filter set to false.

For two Illumina sequenced isolates carrying HCMs and not carrying variant *rpoB*:S450L, LoFreq (Wilm et al., 2012) was used to check for potential subpopulations with that variant by calculating the fraction reads supporting *rpoB*:S450L as a minor variant. These two isolates were not considered to carry *rpoB*:S450L in subsequent analysis, though one of the two carried a potential subpopulation with that variant and was so noted.

VCF formatted files were further annotated with Variant Effect Predictor (VEP) (v87) (McLaren et al., 2016) to determine the consequence of each variant.

### Lineage determination

Lineage for all isolates was determined using a SNP barcode created and implemented by Freschi et al. (2021) in the fast-lineage-caller.

### Phylogeny

4,501 isolates were used to construct a phylogenetic tree, including the 4,309 isolates used in the main analysis. The tree includes the reference strain H37Rv and the outgroup *Mycobacterium canetti* (NC_01995.1). Variants were called and filtered using previously described methods (Modlin et al., 2021). RAxML version 8.2(Stamatakis, 2014) generated the maximum likelihood phylogenetic tree with a general time reversible model and 100 bootstrap replicates. The tree was visualized with the Interactive Tree of Life (Letunic and Bork, 2016) (iToL).

### Identifying putative compensatory mutations

We identified putative compensatory mutations in *rpoA*, *rpoB*, and *rpoC* using the following criteria: (i) the mutation must be carried by at least one RIF^R^ isolate lacking an HCM (Comas et al., 2012); (ii) the mutation must not be carried by any RIF^S^ isolate; (iii) the mutation must be carried by at least two isolates. Four frameshift causing single base deletions were also excluded. To determine whether these mutations were potentially more than bystanders, we then determined whether each novel putative compensatory mutation was carried by an apparently polyphyletic group of isolates using the phylogenetic tree and ETE Toolkit v3.1.1 (Huerta-Cepas et al., 2016). To account for branch ambiguity, isolates on nearby branches were collapsed to count the number of mutation events. These mutation event counts were then used in a Fisher’s exact test of the association between each novel putative compensatory mutation and resistant isolates.

HCMs, previously reported putative compensatory, and novel compensatory mutations were mapped to the *rpoA*, *rpoB*, and *rpoC* genes with known domains using Lollipop (Jay and Brouwer, 2016), with manual adjustments in Inkscape to colors and the heights of mutation labels.

## Results

### Concordance of RIF^R^ genetic markers with phenotypic DST

We analyzed 4,309 whole genome sequences and their phenotypic drug susceptibility testing (DST) results. In total 993 RIF^R^ and 78 RIF^S^ isolates (Table 1; Supplementary Table S1) harbored either a nonsynonymous mutation in the RRDR or one of the confirmed RIF^R^ conferring mutations outside the RRDR (*rpoB*:V170F or *rpoB*:I491F) (World Health Organization, 2021a). Genotype-predicted RIF DST resulted in 92.0% sensitivity and 97.6% specificity. This was slightly lower than the sensitivity (93.8%, confidence interval 93.3–94.2%) and specificity (98.2%, confidence interval 98.0–98.3%) of prediction in a previous study of 27,063 isolates (World Health Organization, 2021a). The lower specificity was likely due to the higher critical concentration use prior to 2021 (Köser et al., 2021). The high critical concentration has been shown to inconsistently classify RIF^R^ for isolates carrying any of six “borderline” mutations in the RRDR that confer lower level RIF^R^ (Supplementary Table S2) (Jo et al., 2017; Köser et al., 2021; World Health Organization, 2021a).

**Table 1:** Number of rifampicin (RIF) resistant and RIF susceptible isolates carrying or not carrying RIF resistance markers.

*rpoB* Genotype	RIF Resistant	RIF Susceptible
No RIF Resistance Marker	86	3,152
Carries any RIF Resistance Marker	993	78
Carries Non-Borderline RRDR Mutation	964	20
Carries Only Borderline RRDR mutation	20	38
Carries V170F or I491F	9	20
Total	1,079	3,230

RIF resistance markers include non-borderline non-synonymous mutations in the rifampicin resistance-determining region (RRDR), borderline mutations in the RRDR, and the *rpoB* mutations V170F or I491F outside the RRDR.

The lower sensitivity may be from sampling bias favoring selection of discordant isolates for whole genome sequencing. The 314 SMRT sequenced isolates had been selected to maximize phenotypic and genotypic diversity from the specimen repositories (Hillery et al., 2014). There were 86 discordant isolates (Table 1), with RIF^R^ DST results despite lacking any non-synonymous RRDR mutation (and lacking *rpoB*:V170F or *rpoB*:I491F). These discordant isolates could be the result of DST error, a resistant subpopulation, an alternative mechanism of resistance, or a false negative genotype call due to lack of coverage. The mean read depth in *rpoB* was 22.1 in RSI sequenced isolates and 132.4 in Illumina sequenced isolates (Supplementary Table S3). To find potential alternative genetic mechanisms of resistance, the 86 discordant RIF^R^ isolates were queried for variants in *rpoA*, *rpoB*, and *rpoC*. Among 42 of the 86 discordant RIF^R^ isolates were 17 unique mutations, of which 7 were exclusive to RIF^R^ isolates (Supplementary Table S4). In the remaining 44 discordant RIF^R^ isolates there were no mutations in *rpoA*, *rpoB*, or *rpoC*. The alternative resistance mechanism in these isolates may through altered gene expression, rather then genotype. Multidrug resistance has previously been observed through altered expression of the ABC efflux pump (Wang et al., 2013). The regulator of this efflux pump, RaaS, may even be under epigenetic regulation, a non-genetic source of diverse gene expression (Modlin et al., 2020).

### High-probability compensatory mutations associated with the common RIF^R^ marker rpoB:S450L

Comas et al. previously identified 12 HCMs in *rpoA* and *rpoC* that likely compensated for the fitness cost of RRDR mutations *in vitro* (Comas et al., 2012). We searched for these HCMs in the 4,309 isolates (Table 2). In total 217 isolates carried an HCMs, including 20.0% (216/1079) of RIF^R^ isolates. Only one isolate with an HCM was RIF^S^. The RIF^S^ isolate carried the common RIF^R^ conferring RRDR mutation *rpoB*:S450L and the HCM *rpoC*:V483G (Supplementary Table S1). The RIF^S^ DST result in this isolate may have been due to the high pre-2021 critical concentration used, however it was more likely a laboratory mix-up, as the isolate carried known resistance markers for 5 drugs (*rpoB*:S450L, *katG*:S315T, *eis*:c-12 t, and *gyrA*:D94N) despite pan susceptible DST results.

**Table 2:** Number of isolates carrying each of the 12 previously identified (Comas et al., 2012) high-probability compensatory mutations (HCMs) in *rpoA* and *rpoC*.

Gene	HCM	Isolates
*rpoA*	T187A	16
*rpoA*	T187P	3
*rpoC*	D485N	21
*rpoC*	I491T	11
*rpoC*	I491V	28
*rpoC*	N698H	2
*rpoC*	N698K	3
*rpoC*	N698S	82
*rpoC*	V483A	16
*rpoC*	V483G	35
*rpoC*	D485H	0
*rpoC*	P434R	0

As reported previously (Song et al., 2014; Borrell and Trauner, 2017; Gygli et al., 2017; Wang et al., 2020; Ma et al., 2021), compensatory mutations were associated with the prevalent RIF^R^ marker *rpoB*:S450L. Isolates carrying *rpoB*:S450L were 48.8 times more likely to carry HCMs than isolates carrying other RIF^R^ markers (Table 3, odds ratio = 48.8, Fisher’s exact test *p* = 7.37e-28). Only two isolates carried an HCM and lacked *rpoB*:S450L. One such isolate carried the non-borderline RRDR mutation *rpoB*:Q432P and the HCM *rpoC*:V483G (Supplementary Table S1). The other isolate carried the RIF^R^ marker *rpoB*:V170F and carried the HCM *rpoA*:T187P (Supplementary Table S1). This isolate also potentially included a subpopulation carrying *rpoB*:S450L. The *rpoB*:S450L variant was supported in the isolate by 7 of the 78 reads mapped to this locus. However, it is uncertain whether these sequencing reads was the result of a genuine subpopulation or sequencing error.

**Table 3:** Number of isolates carrying previously identified (Comas et al., 2012) high-probability compensatory mutations (HCMs) and the common RIF^R^ conferring mutation *rpoB*:S450L.

Compensation Status	Carries *rpoB*:S450L	No *rpoB*:S450L
Carries HCM	215	2
No HCM	587	267

Only isolates carrying RIF^R^ markers are included in these counts.

### Previously reported putative compensatory mutations

We then queried for 175 previously reported putative compensatory mutations from their study and others (Casali et al., 2012, 2014; Comas et al., 2012; de Vos et al., 2013; Song et al., 2014; Li et al., 2016; Liu et al., 2018; Ma et al., 2021). Of these 175 previously reported mutations, we observed 83 mutations across 560 of the 4,309 isolates. In total 555 RIF^R^ and 5 RIF^S^ isolates each carried at least one of these previously reported mutations (Supplementary Table S5). Over half the RIF^R^ isolates (59.1%, 638/1079) carried HCMs or previously reported putative compensatory mutations.

### Novel putative compensatory mutations

**Figure 1:**
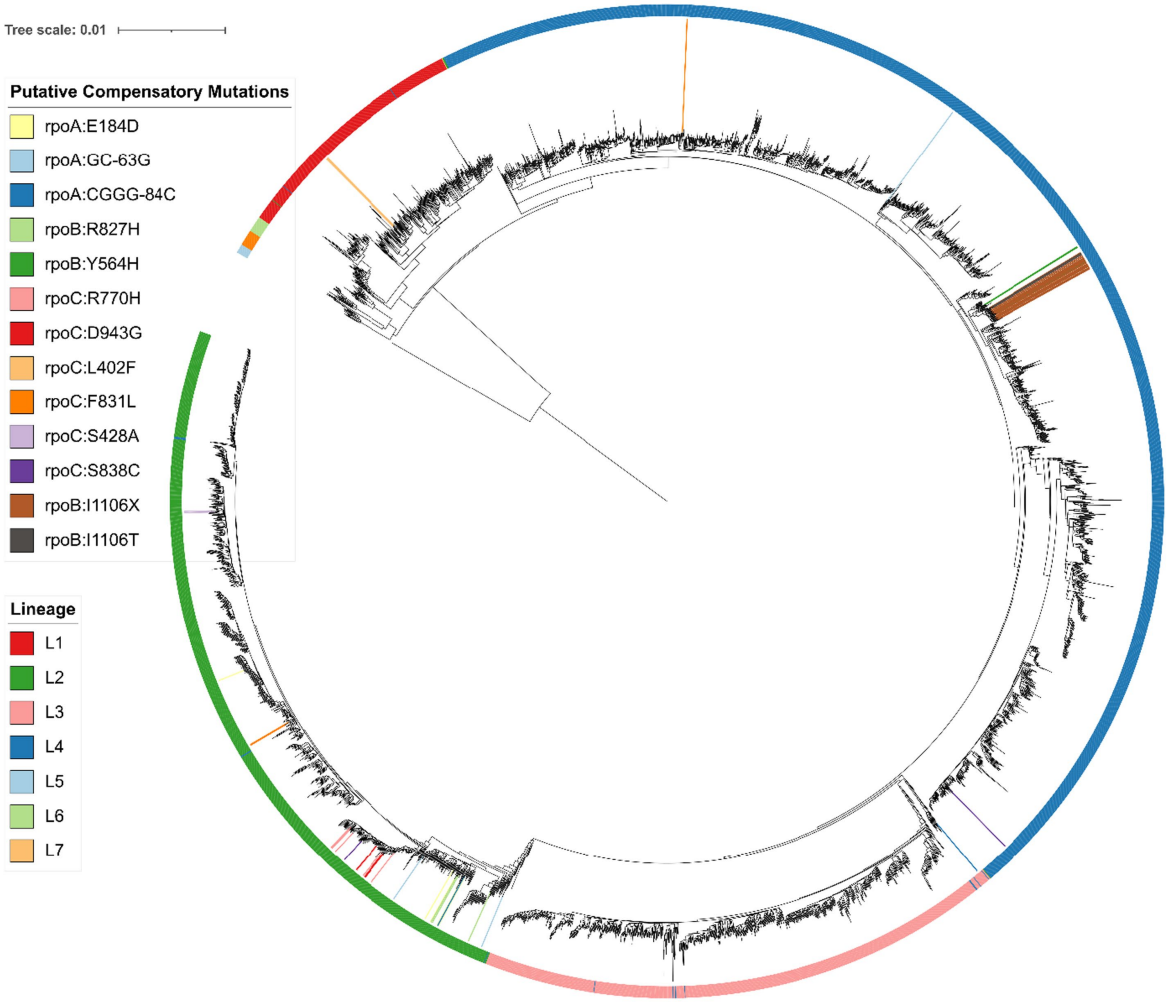
Phylogenetic SNP tree of 4,501 *Mycobacterium tuberculosis* isolates, with *Mycobacterium canettii* as an outgroup, created from variable regions using RAxML (Stamatakis, 2014) and visualized with iTOL (Letunic and Bork, 2016). Branches with a bootstrap support of at least 75/100 are marked with a blue circle. The colored outer ring indicates the lineage of each isolate, as determined using a SNP barcode created and implemented by Freschi et al. (2021) in the fast-lineage-caller. The inner colors indicate the presence of each novel putative compensatory mutation, or the putative compensatory mutation *rpoB*:I1106T (grey) or apparent frameshift *rpoB*:I1106X (brown). The smallest monophyletic group containing the 22 isolates with *rpoB*:I1106T/X includes 34 isolates and has 100/100 bootstrap support. All 34 isolates in this group were collected from the National Health Laboratory Service of South Africa in Johannesburg, South Africa. Suggesting the mutation is inherited from a single mutation event. The most frequent novel putative compensatory mutation, *rpoC*:R770H (pink) is also likely a single mutation event. The smallest monophyletic group that contains these 8 isolates includes 103 isolates and only has 40/100 bootstrap support, with similar ambiguity among the branches within the group. Meanwhile six of the other novel putative compensatory mutations are carried by distant isolates on the phylogenetic tree, indicating two or more mutation events.

We then searched for novel putative compensatory mutations in *rpoABC* with the following criteria: (i) the mutation must be carried by at least one RIF^R^ isolate lacking an HCM (Comas et al., 2012); (ii) the mutation must not be carried by any RIF^S^ isolate; (iii) the mutation must be carried by at least two isolates. We then determined whether each novel putative compensatory mutation was carried by an apparent polyphyletic group of isolates, using a phylogenetic tree (Figure 1). These criteria were developed based on the initial criteria set by Comas et al. (2012) to discover candidate variants, later built upon in Liu et al. (2018), and here modified to use a phylogenetic tree to define relationships among the isolates rather than SNP distance.

These filters identified 11 novel putative compensatory mutations in 37 isolates, of which 10 mutations were apparently polyphyletic (Table 4). The most frequent novel putative compensatory mutation was *rpoC*:R770H, in eight isolates belonging to the Beijing sublineage. The eight isolates grouped closely together on the tree but did not form a monophyletic group (Figure 1), possibly because of branch ambiguity (the smallest monophyletic group containing them had 103 isolates and only 40/100 bootstrap support). These eight isolates all carried the RIF^R^ marker *rpoB*:S450L. The eight isolates may represent a drug resistant outbreak enabled by compensation from *rpoC*:R770H, or may be a mere bystander spread by the outbreak. To account for this branch ambiguity in the tree, isolates on nearby branches were then collapsed together to count the number of mutation events for each novel putative compensatory mutation (Table 4; Figure 1). Six of the novel putative compensatory mutations each had two or more mutation events.

**Table 4:** Number of isolates carrying novel putative compensatory mutations in *rpoA* and *rpoC*.

Gene	Novel putative compensatory mutation	Isolates	Mutation events in phylogenetic tree	Fisher’s Exact Test *p*-value
*rpoC*	R770H	8	1	0.2504
*rpoA*	E184D	6	2	0.06266
*rpoA*	GC-63G	3	3	0.01567
*rpoB*	R827H	3	2	0.06266
*rpoB*	Y564H	3	1	0.2504
*rpoC*	D943G	3	1	0.2504
*rpoC*	L402F	3	1*	0.2504
*rpoC*	F831L	3	2	0.06266
*rpoA*	CGGG-84C	2	2	0.06266
*rpoC*	S428A	2	1	0.2504
*rpoC*	S838C	2	2	0.06266

Each reported mutation was carried by at least two isolates, was carried exclusively by RIF^R^ isolates, and was carried by at least one RIF^R^ isolate lacking a previously identified (Comas et al., 2012) high-probability compensatory mutation. Note that four putative novel compensatory mutations were excluded (*rpoB*:D435X, *rpoC*:E1137X, *rpoC*:P390X, *rpoC*:V483X) because they were frameshifts, and in the essential *rpoABC* genes frameshifts are likely the result of errors in sequencing or variant calling. A phylogenetic tree was used to determine the apparent number of mutation events for each mutation, collapsing isolates on nearby branches. These counts were used in a Fisher’s exact test of the association between each novel putative compensatory mutation and RIF^R^ isolates. The mutation with an asterisk in the Mutation Events column was called *Monophyletic using ETE Toolkit v3.1.1.

Together 62.1% (670/1079) of RIF^R^ isolates carried either an HCM, a previously reported putative compensatory mutation, or a novel putative compensatory mutation. Most of the isolates with novel mutations carried *rpoB*:S450L (Table 5). Just as with HCMs and as reported previously (Song et al., 2014; Borrell and Trauner, 2017; Gygli et al., 2017; Wang et al., 2020; Ma et al., 2021), compensatory mutations associated *rpoB*:S450L. Including novel mutations, isolates carrying *rpoB*:S450L were 18.4 times more likely to carry an HCM or putative compensatory mutation than isolates carrying other RIF^R^ markers (Supplementary Table S7, odds ratio = 18.4, Fisher’s exact test *p* = 1.18e-74).

**Table 5:** Number of isolates carrying rifampicin resistance markers common in the dataset.

Marker	R	S	HCM	Reported Putative Comp	Novel Putative Comp
S450L	801	1	215	515	35
D435V	40	2	1	2	1
L452P	32	10	0	19	0
H445Y	31	2	0	1	0
D435G	24	1	0	17	0
H445D	21	1	0	1	0
I491F	6	20	0	1	0
S450L D435V	3	0	1	2	0
S450L H445Y	1	0	0	1	0
S450L H445D	1	0	0	0	0
L452P H445Y	0	1	0	0	0
L452P D435G	16	0	0	13	0

All RRDR mutations carried by at least 20 isolates are included, as well as the known rifampicin resistance marker *rpoB*:I491F. Columns “R” and “S” report the number of rifampicin resistant and susceptible isolates carrying each mutation, according to phenotypic drug susceptibility testing. Column “HCM” reports the number of isolates carrying any of the 12 high-probability compensatory mutations previously reported by Comas et al. (2012). Column “Reported Putative Comp” reports the number of isolates carrying both the indicated resistance marker and any previously reported (Casali et al., 2012, 2014; Comas et al., 2012; de Vos et al., 2013; Li et al., 2016; Liu et al., 2018) putative compensatory mutations. Column “Novel Putative Comp” reports the number of isolates carrying both the indicated resistance marker and any of the novel putative compensatory mutations identified in this study. The last five rows report the number of isolates carrying combinations of multiple of the markers from the table. Combinations carried by zero isolates are excluded.

This method also independently corroborated 35 previously reported (Casali et al., 2012, 2014; Comas et al., 2012; de Vos et al., 2013; Song et al., 2014; Li et al., 2016; Liu et al., 2018; Ma et al., 2021) putative compensatory mutations (Supplementary Table S6). One such independently identified putative compensatory mutation, *rpoB*:I480V, was recently confirmed by mutagenesis and competitive fitness assays to compensate in *M. smegmatis in vitro* (Ma et al., 2021). These independently identified putative compensatory mutations identified by this method supports the reproducibility and validity of the method.

One noteworthy independently identified putative compensatory mutation was *rpoB*:I1106T. This mutation was previously reported in two extremely drug resistant (XDR) isolates from South Africa from a suspected outbreak, each with the pair of RIF^R^ markers *rpoB*:D435G and *rpoB*:L452P (Song et al., 2014). We also observed these three mutations together in two isolates from South Africa, possibly from the same outbreak as the previous study. There was also evidence of these three mutations in 20 more closely related isolates from South Africa, further corroborating that these three mutations were carried by an outbreak. These 20 closely related isolates had the variant call *rpoB*:I1106X (Figure 1). This suggests that *rpoB*:I1106X may have been a systematically miss-called variant indicating the same underlying mutation as *rpoB*:I1106T. At the nucleotide level, the SNP *rpoB*:I1106T (T3317C) extended a homopolymer (from ATCCCG to ACCCCG). The 20 isolates with *rpoB*:I1106X were SMRT sequenced with P4C2 chemistry and variant called with PBHoover (Ramirez-Busby et al., 2018), while the two isolates with *rpoB*:I1106T were SMRT sequenced with P6C4 chemistry and variant called with mummer (Kurtz et al., 2004) after *de novo* assembly. The PacBio RS platform with P4C2 chemistry has a significant error bias towards single base insertions and deletions, and homopolymers only exacerbate this error bias(Ross et al., 2013).

The 22 isolates with *rpoB*:I1106T/X were all RIF^R^ and each carried two RRDR mutations at the codons 452 and 435 (*rpoB*:D435G or *rpoB*:D435X and *rpoB*:L452P or *rpoB*:L452X), the same RIFR markers reported in the two isolates previously reported (Song et al., 2014) with *rpoB*:I1106T. These rare RRDR mutations were carried in only 33 isolates. Isolates carrying *rpoB*:I1106T/X were at least 663 times more likely to carry either *rpoB*:D435G/X or *rpoB*:L452P/X than isolates without *rpoB*:I1106T/X (Supplementary Table S8, odds ratio 95% confidence interval: 663 to ∞, value of *p* = 1.71e-44). The rare RIF^R^ markers *rpoB*:D435G and *rpoB*:L452P may have been able to cause the resistant outbreak despite a high fitness cost because of the previously reported (Song et al., 2014) putative compensatory mutation *rpoB*:I1106T.

**Figure 2:**
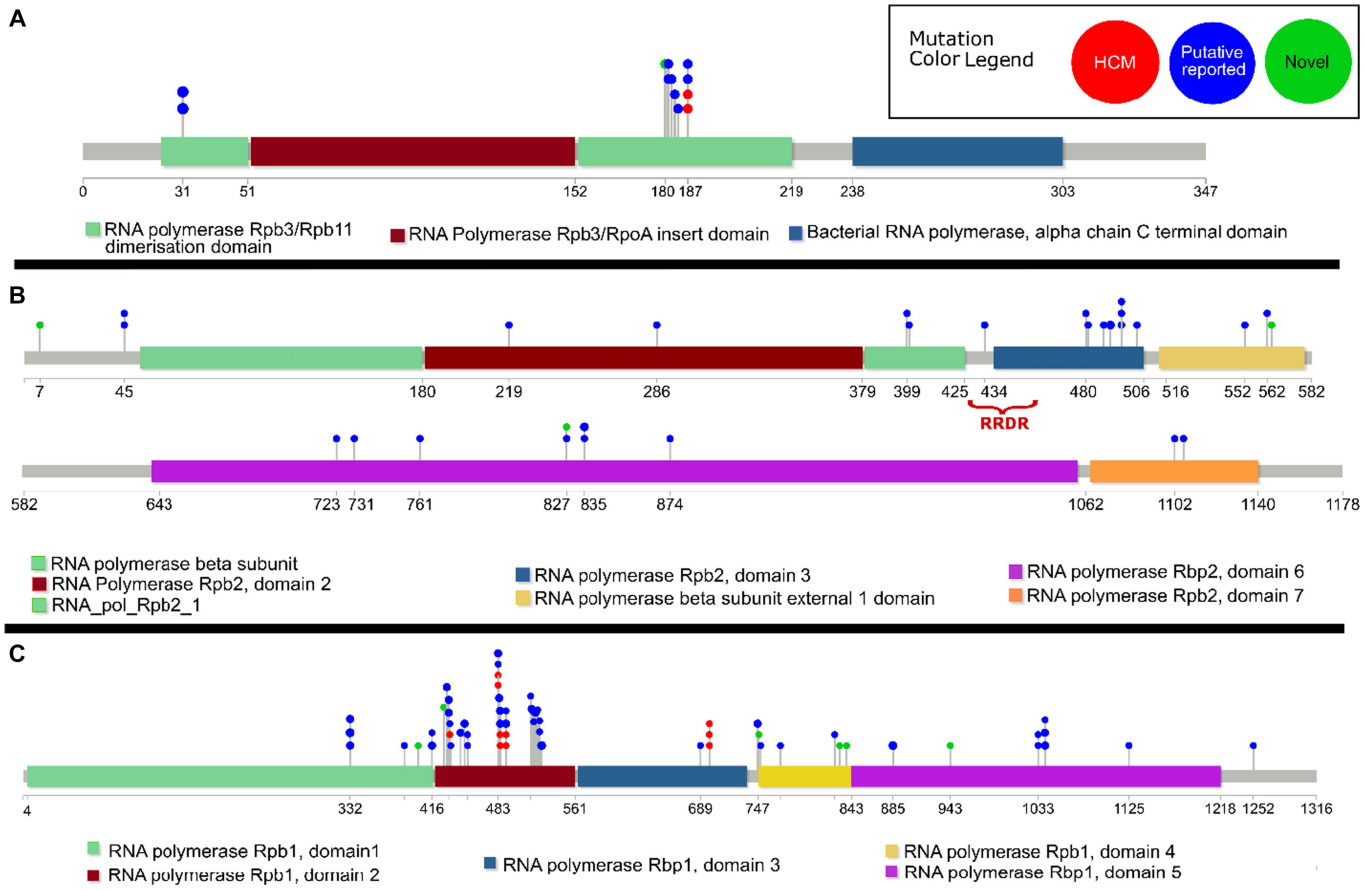
Lollipop diagram of previously reported^14^ high-probability compensatory mutations (“HCM”), previously reported (Casali et al., 2012, 2014; Comas et al., 2012; de Vos et al., 2013; Song et al., 2014; Li et al., 2016; Liu et al., 2018; Ma et al., 2021) putative compensatory mutations (“Putative reported”), and novel putative compensatory mutations (“Novel”) in the *M. tuberculosis* genes **(A)** *rpoA*, **(B)** *rpoB*, and **(C)** *rpoC*. The functional domains of each gene are indicated, as well as the rifampicin resistance determining region (RRDR) in *rpoB*. Only previously reported putative compensatory mutations carried by isolates in this dataset are included.

HCMs and putative compensatory mutations in *rpoA* were exclusively in the RNA polymerase Rpb3/Rpb11 dimerization domain (Figure 2A). In *rpoC* the HCMs and putative compensatory mutations primarily clustered in Domain 2 (Figure 2C). In *rpoB*, there was a higher concentration of putative compensatory mutations in domain 3 (Figure 2B).

## Discussion

Resistance to RIF is prevalent despite the fitness cost of RIF^R^ conferring mutations. Multiple RIF^R^ mutations in *rpoB* have been shown to reduce growth rate *in vitro* (Gagneux et al., 2006; Comas et al., 2012) and in macrophages (Mariam et al., 2004). Yet rifampicin resistant strains continue to spread, and in some countries constitute an increasing threat to an effective TB control (World Health Organization, 2020). One potential explanation for this discrepancy is compensatory mutations in *rpoA*, *rpoB*, and *rpoC*. Three mutations in these genes have been observed in clinical RIF^R^ *M. tuberculosis* isolates with a restored wildtype growth rate *in vitro* (Comas et al., 2012). Two compensatory mutations have been shown to restore transcription efficiency to ribosomes with the RIF^R^ marker rpoB:S450L (Stefan et al., 2018). Many more mutations in these genes have been associated with clinical RIF^R^ strains (Comas et al., 2012; de Vos et al., 2013; Song et al., 2014; Li et al., 2016; Liu et al., 2018). And five mutations in *rpoB* have recently been confirmed by mutagenesis and competitive fitness assays to compensate for *rpoB*:S450L in *M. smegmatis* (Ma et al., 2021). RIF^R^ strains with compensatory mutations may have higher transmission rates than RIF^R^ strains without them (de Vos et al., 2013; Casali et al., 2014; Li et al., 2016; Merker et al., 2018; Gygli et al., 2021), though this is disputed in at least one study (Liu et al., 2018). Compensatory mutations may thus serve as markers warning which resistant strains are more likely to cause outbreaks. We expanded the set of putative compensatory mutations by examining *rpoABC* mutations in 4309 whole genome sequenced clinical *M. tuberculosis* isolates. Based on our findings we also suggest the hypothesis that each compensatory mutation may only compensate for a specific resistance-conferring mutation, rather than all compensatory mutations compensating for all resistance-conferring mutations. If confirmed, these findings will improve accuracy in determining which strains are at higher risk of causing drug resistant outbreaks.

Previously reported compensatory mutations (Casali et al., 2012, 2014; Comas et al., 2012; de Vos et al., 2013; Song et al., 2014; Li et al., 2016; Liu et al., 2018; Ma et al., 2021) were observed in over half the RIF^R^ isolates (59.1%, 638/1079). Using strict criteria, we identified 11 novel putative compensatory mutations in 37 of the remaining isolates (Table 4). Six of these 11 novel putative compensatory mutations had two or more mutation events. Our criteria additionally independently identified 35 of the previously reported putative compensatory mutations (Supplementary Table S6). One of these independently identified mutations, *rpoB*:I480V, was confirmed by mutagenesis to compensate for *rpoB*:S450L *in vitro* in *M. smegmatis* (Ma et al., 2021). This independent corroboration of 35 putative compensatory mutations identified by our method supports the validity of the 11 novel putative compensatory mutations identified by our criteria. These 11 novel putative compensatory mutations, together with those previously reported, may serve as markers to warn clinicians which RIF^R^ strains are more likely to cause outbreaks.

Another potential barrier to identifying compensated strains is the specificity of compensatory mutations. Only three RIF^R^ markers were associated with compensatory mutations: *rpoB*:S450L, *rpoB*:L452P, and *rpoB*:D435G (Tables 3, 5; Supplementary Tables S6, S8). Most compensatory mutations were associated with the prevalent RRDR mutation *rpoB*:S450L, an association reported previously (Song et al., 2014; Borrell and Trauner, 2017; Gygli et al., 2017; Wang et al., 2020; Ma et al., 2021). However the previously reported (Song et al., 2014) putative compensatory mutation *rpoB*:I1106T was specific to isolates with a pair of rare RRDR mutations, *rpoB*:L452P and *rpoB*:D435G. These three mutations were each carried by 22 closely related isolates (Figure 1), suggesting the putative compensatory mutation overcame the fitness cost of the otherwise rare RIF^R^ markers, enabling them to cause a resistant outbreak. We hypothesize that *rpoB*:L452P and *rpoB*:D435G are normally rare because only *rpoB*:I1106T can compensate for their fitness cost, while most other compensatory mutations compensate for *rpoB*:S450L. If each compensatory mutation only compensates for specific resistance-conferring marker, then predicting which RIF^R^ strains have higher risk of causing outbreaks requires not only a catalogue of compensatory mutations, but knowledge of which combinations of *rpoABC* mutations compensate for each other.

A recent study of 27,063 isolates found *rpoB*:S450L in 66.2% of RIF^R^ isolates (6,536/9869) (World Health Organization, 2021a). Our findings suggest that the high prevalence of *rpoB*:S450L among RIF^R^ isolates (both in this, and previous *in vitro* and *in vivo* (Huitric et al., 2006) studies) is likely due to two factors. First, as previously reported, this variant has a lower fitness cost as compared to other resistant-causing variants (Gagneux et al., 2006). Second, as our findings indicate, mutation-specific-compensation may also be responsible for the prevalence of *rpoB*:S450L. In our findings most compensatory mutations, identified previously or through this study, appear to only compensate only for *rpoB*:S450L. Pengjiao Ma et al.’s findings support our dual reasoning hypothesis for prevalence of *rpoB*:S450L (Ma et al., 2021).

The clear next steps for the hypotheses generated through this study is mutagenesis and competitive fitness experiments to confirm the compensatory role. This includes the 11 novel putative compensatory mutations that this study has identified. Mutagenesis and competitive fitness is also needed to test the mutation-specific-compensation hypothesis suggested by our findings, by testing whether compensatory mutations specifically compensate for *rpoB*:S450L, or other RIF^R^ mutations. Additionally, the criteria to identify these novel putative compensatory mutations was conservative and thus our expanded set of compensatory mutations is more reliable, but not comprehensive. The study also used a limited set of isolates (4309) from a limited set of world regions (9 countries), and would thus miss any region specific or rare mutations. Further study is needed to determine the full set of compensatory mutations.

This study identified 11 novel *rpoABC* mutations that putatively compensate for the fitness cost of RIF^R^ mutations. This study also independently corroborated another 35 previously reported (Casali et al., 2012, 2014; Comas et al., 2012; de Vos et al., 2013; Song et al., 2014; Li et al., 2016; Liu et al., 2018; Ma et al., 2021) putative compensatory mutations. These mutations may aid future investigation of the effect of compensatory mutations on RIF^R^ TB strain transmission, and eventually aid the detection of strains at high risk of causing RIF^R^ outbreaks. This study additionally found that compensatory mutations were associated with specific RIF^R^ markers, corroborating the previously reported (Song et al., 2014; Borrell and Trauner, 2017; Gygli et al., 2017; Wang et al., 2020; Ma et al., 2021) association between compensatory mutations and rpoB:S450L and greatly expanding the previously reported (Song et al., 2014) outbreak with the putative compensatory mutation *rpoB*:I1106T and the RIF^R^ markers *rpoB*:D435G and *rpoB*:L452P. These associations highlight the need for future study of which combinations of RIF^R^ markers and *rpoABC* mutations result in compensation.

## Data availability statement

The datasets presented in this study can be found in online repositories. The names of the repository/repositories and accession number(s) can be found in the article/Supplementary material.

## Author contributions

DC-G: Conceptualization, Investigation, Methodology, Validation, Writing – original draft, Writing – review & editing, Data curation, Formal analysis, Software, Visualization. SR-B: Conceptualization, Formal analysis, Investigation, Methodology, Validation, Visualization, Writing – review & editing. BG: Data curation, Formal analysis, Software, Visualization, Writing – original draft, Writing – review & editing. AE: Conceptualization, Formal analysis, Methodology, Software, Visualization, Writing – original draft, Writing – review & editing. SH: Conceptualization, Investigation, Writing – review & editing. WE: Conceptualization, Investigation, Methodology, Writing – review & editing. FV: Conceptualization, Funding acquisition, Investigation, Methodology, Project administration, Resources, Supervision, Validation, Writing – original draft, Writing – review & editing.

## Footnotes

^1^ https://gitlab.com/LPCDRP/mummer-extras/-/blob/master/src/mummer-snps2vcf
